# Tissue RNA Sequencing Reveals Novel Biomarkers Associated with Postoperative Keloid Recurrence

**DOI:** 10.3390/jcm12175511

**Published:** 2023-08-25

**Authors:** Yanqiu Tang, Kehui Ren, Xufeng Yin, Yunning Yang, Fang Fang, Bingrong Zhou, Wenbo Bu

**Affiliations:** 1 Department of Dermatology, The First Affiliated Hospital of Nanjing Medical University, Nanjing 210029, China; tyq1026@163.com (Y.T.); r1427474708@163.com (K.R.); xu13010313@163.com (X.Y.); yyn133456@163.com (Y.Y.); 2 Department of Dermatologic Surgery, Dermatology Hospital of Chinese Academy of Medical Sciences, Nanjing 210042, China; fangfangjh@126.com

**Keywords:** keloid, surgery, recurrence, RNA-Seq, gene, WGCNA

## Abstract

Keloids can be resected through surgery, but they may still recur. The purpose of this study was to explore the biomarkers to predict the postoperative recurrence of keloids. Patients who underwent surgical treatment and postoperative superficial X-ray radiation between January 2019 and December 2020 were recruited with clinical data and keloid samples for RNA-seq. By screening differentially expressed genes (DEGs) between postoperative recurrent and non-recurrent sample groups and constructing a co-expression network via the weighted gene co-expression network analysis (WGCNA), an immunity-related module was chosen for subsequent analysis. By constructing a DEG co-expression network and using the Molecular Complex Detection (MCODE) algorithm, five hub genes were identified in the key module. Receiver Operating Characteristic (ROC) curve analysis showed that the area under the curve (AUC) for the five combined hub genes was 0.776. The result of qRT-PCR showed that *CHI3L1*, *IL1RN*, *MMP7*, *TNFAIP3,* and *TNFAIP6* were upregulated in the recurrent group with statistical significance (*p* < 0.05). Immune infiltration analysis showed that mast cells, macrophages, and T cells were the major components of the keloid immune microenvironment. This study provides potential biomarkers for predicting keloid recurrence and offers insights into genetic targets for recurrence prevention.

## 1. Introduction

Keloids are a fibroproliferative disorder, characterized by abnormal proliferation of fibroblasts and excessive deposition of extracellular matrix (ECM) [1]. Usually benign, they may still grow aggressively beyond their original boundary, causing deformity, itching, pain, and functional or even psychological disorders [1]. The pathophysiological mechanisms behind it have not been fully elucidated, but they have been shown to involve macrophages, lymphocytes, mast cells, and other immune cells [2,3]. Treatments mainly include surgery, adjuvant radiation, laser, and steroid injections [4]. However, their outcomes remain unsatisfactory, as indicated by frequent recurrence [5]. No consensus has been reached regarding the management of postoperative keloid recurrence [5]. For patients who are prone to relapse after surgery, early intervention is a major option [4]. 

Infiltrative borders (borders not clearly demarcated and not easily defined) of keloids are accompanied by a recurrence rate enhancing one year [6]. Residual keloid tissues after surgical resection behave like invasive tumors, which may induce the postoperative recurrence of keloids [7]. Nangole et al. revealed a positive correlation between keloid recurrence rate and the absolute counts of lymphocytes and macrophages [8]. Cytokines in Th2 cells, especially interleukin (IL)-4, are overexpressed in keloid tissues [9]. Meanwhile, dupilumab, an IL-4 receptor antagonist, has been reported to treat keloids effectively [10]. In the pathogenesis of postoperative keloid recurrence, inflammation and immunity may play an essential role.

High-throughput RNA sequencing (RNA-Seq) is an effective strategy to explore therapeutic targets in keloids [9]. Min et al. proposed that the inhibition of the *FOXO1* (Forkhead box O1) pathway, as a novel strategy, may radiosensitize keloids, according to their mRNA sequencing analysis illustrating the molecular changes in fibroblasts after exposure to radiation in keloids [11]. A recent study found that fibroblast activation protein (*FAP*) alpha was significantly overexpressed in recurring keloids after surgical resection and radiation therapy [12]. Weighted co-expression network analysis (WGCNA) has been used to explore genes related to the progression of keloids [13]. For example, Liu et al. revealed aberrant gene expression in keratinocytes and fibroblasts in keloids and used these genes to construct regulatory networks [14]. Using single-cell RNA sequencing and WGCNA, Xie et al. screened *TNC* (Tenascin-C) as a biomarker of keloids [15]. WGCNA is an algorithm used to identify similar expression patterns based on gene expression levels. Unlike WGCNA, DEG analysis focuses on the differences between samples, whereas WGCNA focuses on the relationships between genes. WGCNA distinguishes genes into certain modules by analyzing the similarities among genes. By analyzing the correlations between different modules and certain clinical phenotypes, it is possible to identify the molecular characteristics associated with specific clinical phenotypes. Despite these advances, the molecular characteristics of keloid recurrence require more investigation. In this study, we combined several informatics tools, including WCGNA, to identify biomarkers that can predict postoperative recurrence in 77 patients with keloids.

## 2. Methods

### 2.1. Study Design and Patients

Tissue samples and clinical data were obtained from patients who underwent surgical resection at the Institute of Dermatology, Chinese Academy of Medical Sciences (Nanjing, China) from January 2019 to December 2020. The ethics committee of the Chinese Academy of Medical Science and Peking Union Medical College approved this study (No. 2017-ky-006). The patients provided written informed consent to participate in this study in accordance with the Declaration of Helsinki. The inclusion criteria for patients were as follows: (1) keloids were diagnosed by two dermatologists based on clinical (lesions extending beyond the original injury site, invading the surrounding normal skin, and showing symptoms such as redness, itchiness, and pain) and pathological features (epidermal thickening, overproliferation of fibroblasts, and accumulation of ECM); (2) the patient received surgical resection; (3) the patients’ follow-up and baseline data were complete. The exclusion criteria were as follows: (1) the patient underwent laser, cryotherapy, or additional surgical intervention six months before, during, and 12 months after surgery; (2) the patient received intralesional treatments, such as steroids or chemotherapeutics, 6 months before the surgery. 

### 2.2. Surgical Treatment Regimen and Follow-Up Protocol

All patients received conventional examination after admission to the hospital. The Vancouver scar scale (VSS) and the Dermatology Life Quality Index (DLQI) were used to evaluate the status of keloids before surgery. All keloid tissues were removed by surgical resection. Most of the patients underwent superficial X-ray radiation within one week after surgery (20 Gy in 4–5 fractions, SRT-100, Sensus Healthcare, Boca Raton, FL, USA). In some cases, gene expression patterns may vary in different keloid parts. To avoid the effect of this heterogeneity on prognosis, we only collected the keloid tissues with an obvious inflammatory appearance after surgical resection for subsequent RNA-seq detection. If there was no significant heterogeneity in keloid tissues, we randomly selected a portion for detection. The tissues were frozen in liquid nitrogen and stored at −80 °C for further RNA isolation. All patients were followed for at least 1 year. We analyzed the patients’ clinical features, including age, disease course, sex, site of onset, etiology, postoperative radiotherapy, pre-operative VSS, and DLQI scores. Recurrence was defined as keloids re-growing beyond the excision margins or patients demonstrating pruritus or pain more serious than those before the resection during the follow-up period [8].

### 2.3. RNA Extraction and Library Construction

The detailed protocol for extracting RNA and constructing a library can be found in the Supplementary Explanation.

### 2.4. Quality Control and DGE Analysis

The data generated via high-throughput sequencing underwent conversion into sequence data using the CASAVA base calling method. The outcomes were then saved in a FASTQ format file containing detailed information about the sequence of reads and their corresponding quality scores. The raw data in FASTQ format were processed using in-house Perl scripts. To extract clean reads, low-quality reads containing adapters and reads with N bases were excluded from the following analysis. All subsequent analyses were based on these high-quality genes. A total of 53,735 genes were identified in this study. Differential expression analysis between the recurrence and non-recurrence groups was performed with the R package DeSeq2 (version 1.12.0). The *p*-values were calculated using Benjamini and Hochberg’s method. Genes that exhibited a *p*-value less than 0.05 and an absolute log2 fold change (|log2FC|) greater than 1.5 were classified as differentially expressed genes (DEGs).

### 2.5. Principal Component Analysis (PCA)

PCA was performed on the DEGs of all samples to identify the ability of group segregation [16]. The *p* value was derived from the analysis of variance (ANOVA) of the first principal component between the two groups.

### 2.6. WGCNA

The WGCNA [17] was used to construct a co-expression network of all genes associated with keloid recurrence. First, Pearson correlation matrices were conducted for all genes. Second, the adjacency matrix was represented by a similarity matrix, which was specifically depicted as a_i, j_ = |Cor (X_i_, X_j_)|^β^ (where β denotes the soft threshold). Finally, a topological matrix (TOM) was calculated based on the adjacency matrix to detect gene modules. The gene co-expression modules were displayed using a dynamic tree-cut algorithm [18]. The number of genes in the module was more than 30, and the branch merge was cut by 0.25.

### 2.7. Functional and Pathway Enrichment Analyses

To explore gene functions, Gene Ontology (GO) and Encyclopedia of Genes and Genomes (KEGG) enrichment analyses of the DEGs were performed using the ClusterProfiler package 3.4.4. Enrichment significance was defined as a *p*-value less than 0.05. The R package named “ggplot2” was used to visualize the results of GO and KEGG analysis. 

### 2.8. Identification of Key Gene Modules

The functions of the DEGs were annotated, and the key module containing DEGs with similar functions was selected for subsequent analyses. 

### 2.9. Construction of DEG Co-Expression Network and Identification of Hub Genes

The STRING database (http://string-db.org/; accessed on 5 March 2023) was used to directly map the DEGs into a co-expression network of key modules; then, the co-expression network based on the DEGs in the key module was constructed and visualized by Cytoscape v3.8.2. Highly connected sub-networks of the DEG co-expression network were analyzed using the MCODE plug-in. Hub genes were identified as the top 5 nodes in the DEG co-expression network. Using the pROC package (3.6.3), the ROC curve was plotted, and the AUC value was calculated to evaluate the ability of hub genes to predict the recurrence of keloids. 

### 2.10. qRT-PCR Validation for Hub Genes

Ten samples independent of those in RNA-seq were collected (5 recurrence and 5 non-recurrence) for qRT-PCR validation. The expression profiles of 5 hub genes considered to be possible biomarkers were validated in tissue samples by qRT-PCR. An RNA extraction kit (G3013, Servicebio) was used to finely ground keloid tissues to extract total RNA. RNA was reverse transcribed into cDNA using Servicebio^®^RT First Strand cDNA Synthesis Kit (G3330, Servicebio). Subsequently, qRT-PCR was performed using 2×SYBR Green qPCR Master Mix (G3320, Servicebio), according to the manufacturer’s instructions. All primer oligos were synthesized by Servicebio (Wuhan, China) (Table S1).

### 2.11. Immune Infiltration Analysis

The relative immune infiltration levels of the 22 cell types in all samples were computed using the CibersortX package and the reference “LM22.txt” [19]. Spearman correlation analyses were performed to assess the relationship between the expression of hub genes and the infiltration of immune cells. The results were visualized using the ggplot2 package. 

### 2.12. Statistical Analysis

All the statistical analyses were carried out using R software (4.1.1). Continuous data were expressed as median ± standard deviation. Categorical variables were expressed as numbers and percentages. Univariate and multivariate analyses were used to identify the risk factors for keloid recurrence. Each risk factor was described using odds ratios (ORs) with 95% CI. The ROC curves were compared using the DeLong test. ROC analysis based on the multivariate logistic regression model was conducted to assess the diagnostic value of the combined assays. 

## 3. Results

### 3.1. Baseline Patient Information and Clinical Prognostic Factors

**Figure 1:**
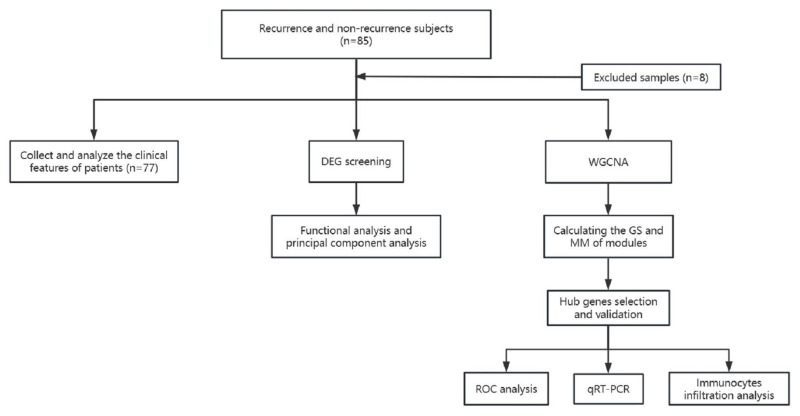
Flowchart of the study.

The study flowchart is shown in Figure 1. A total of 77 patients were included, with relapse occurring in 7 patients after 1 year (a relapse rate of 9.1%). Statistically significant differences were observed in sex (male: 100.0% vs. 50.0% (non-recurrence vs. recurrence); female: 0.0% vs. 50.0% (non-recurrence vs. recurrence), *p* = 0.011) and etiology (surgery: 14.3% vs. 17.1% (non-recurrence vs. recurrence); trauma: 0.0% vs. 10.0% (non-recurrence vs. recurrence; inflammation: 85.7% vs. 24.3% (non-recurrence vs. recurrence); unknown: 48.6% vs. 0.0% (non-recurrence vs. recurrence), *p* = 0.007) between recurrence and non-recurrence groups. Univariate and multivariate regression analyses revealed no significant correlation between postoperative recurrence of keloids and sex, age, or etiology. The results are shown in Table 1.

### 3.2. DEGs Identified by RNA-seq

**Figure 2:**
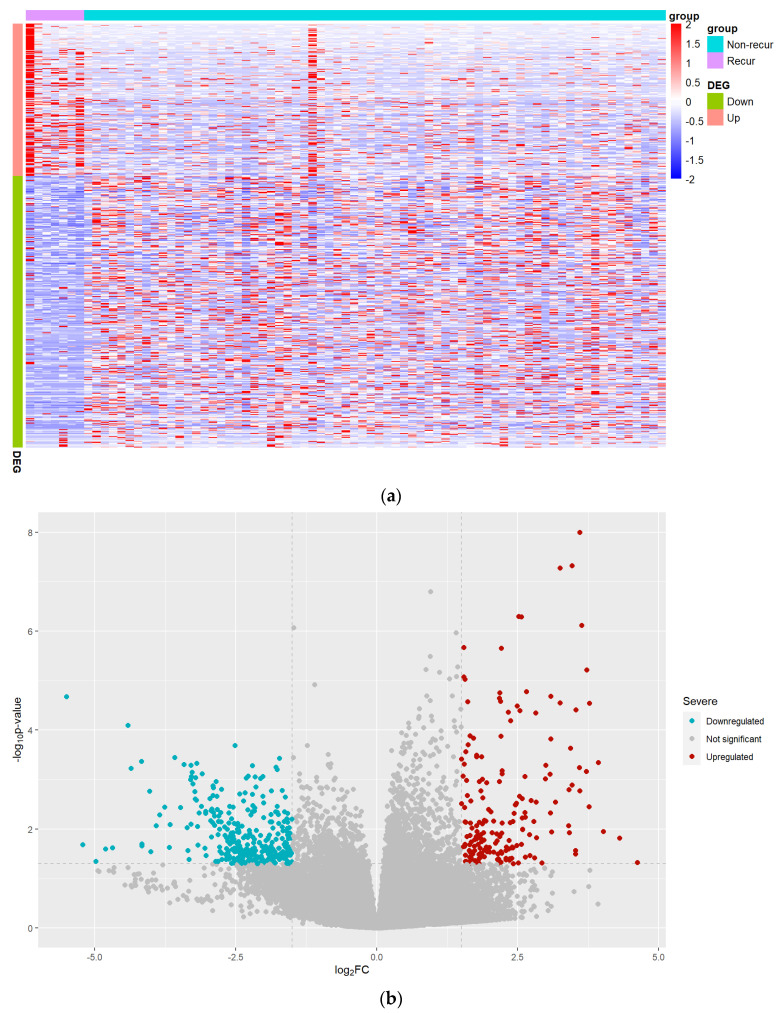
DEGs between recurrent and non-recurrent samples. (**a**) Heatmap of gene expression. The heatmap showed the expression pattern of genes. (**b**) Volcano plot of differential genes. The x-axis represents log2 (fold−change) while the y-axis represents −log10 (*p* value). The red dots represent upregulated DEGs while the green dots represent downregulated DEGs.

Differential gene expression heat map in keloid tissue is displayed in Figure 2a. Differential expression analysis revealed a list of upregulated and downregulated DEGs (Table S2). In total, 534 DEGs were identified, including 192 upregulated and 342 downregulated (Figure 2b). Table 2 shows the top 10 upregulated and downregulated *DEGs*. PCA of all DEGs showed a slight distinction between the recurrence and non-recurrence groups (*p* < 0.001, Figure S1).

### 3.3. Co-Expression Network Constructed by WGCNA

**Figure 3:**
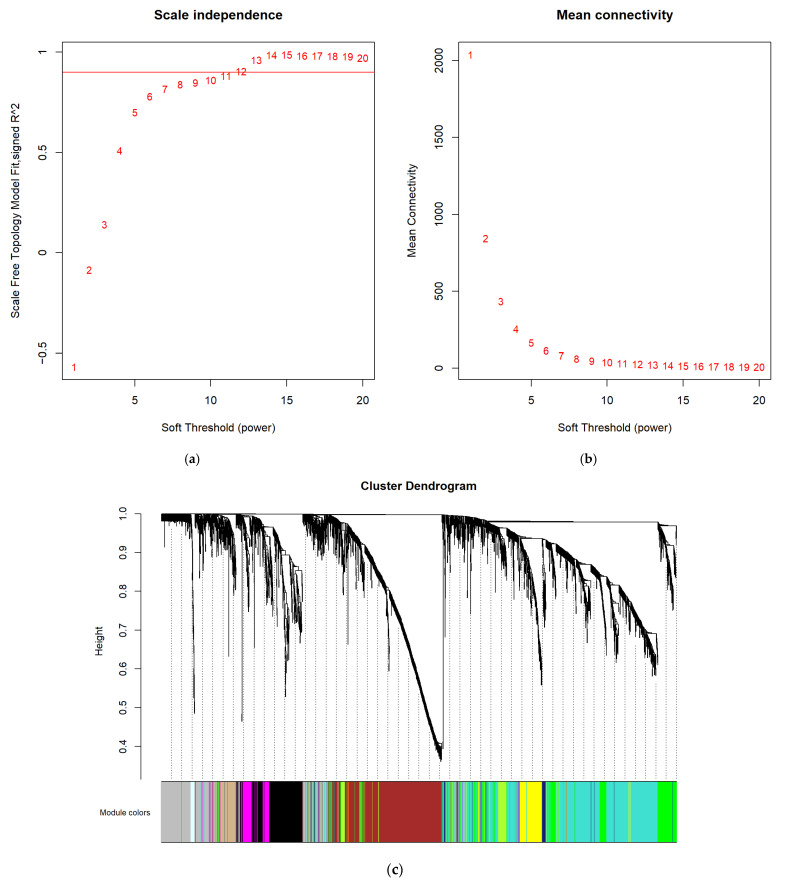

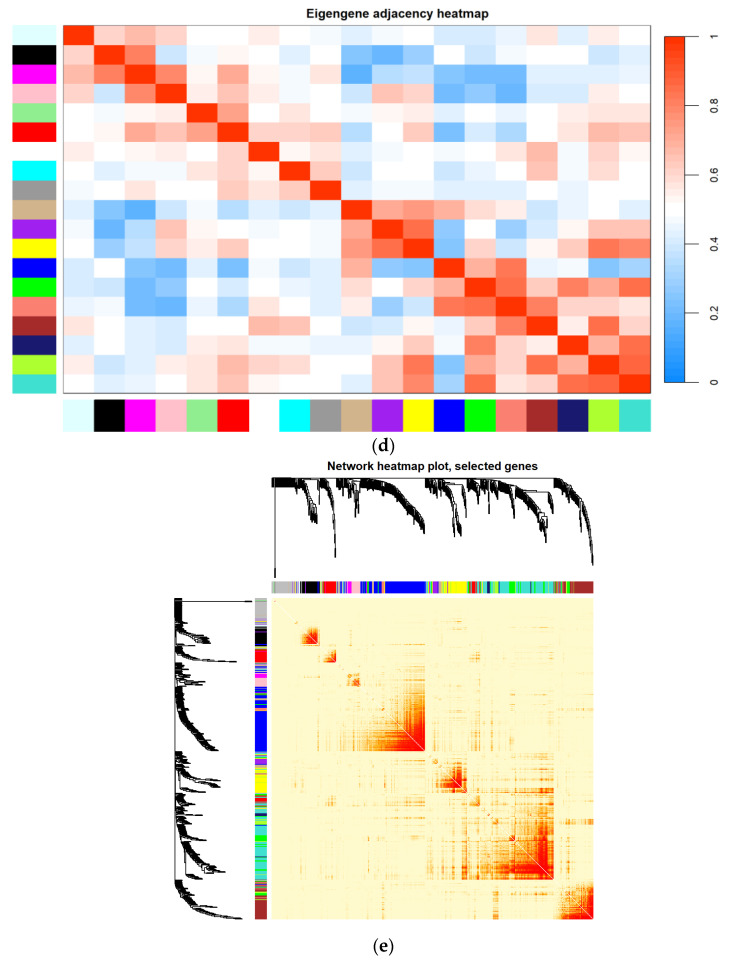
Construction of the co-expression network. (**a**,**b**) Construction of the scale-free network with a suitable soft-thresholding power (β). The red line represents the value of the scale-free fit index (0.80). (**c**) Cluster dendrogram. Each branch represents a module. The original and merged modules are shown in the two-color bars below. (**d**) Eigengene adjacency heatmap. The colors of the squares from red to blue indicate the high-to-low adjacency of the corresponding modules. (**e**) Heatmap plot of all genes. The colors from light to deep red represent a low-to-high interaction. The left and top of the figure show the gene dendrogram and module allocation.

We selected β = 12 to construct a scale-free network (Figure 3a,b). A total of 18 co-expressed modules were detected using the dynamic tree-cut approach (Figure 3c). The number of genes in each module ranged from 23 to 1427. Genes in the grey module failed to be classified into any other module. Therefore, the enrichment analysis was not conducted based on the genes in the grey module. The correlation between modules was demonstrated using the module eigengene adjacency heatmap (Figure 3d). Most modules showed a low adjacency to others, indicating that the module was independent and precise. The network heatmap displaying the correlations between all genes is presented in (Figure 3e). The genes in the same module were highly intercorrelated but weakly correlated with those in the other modules. Thus, the reliability of the modules was verified.

### 3.4. Results of Enrichment Analyses

**Figure 4:**
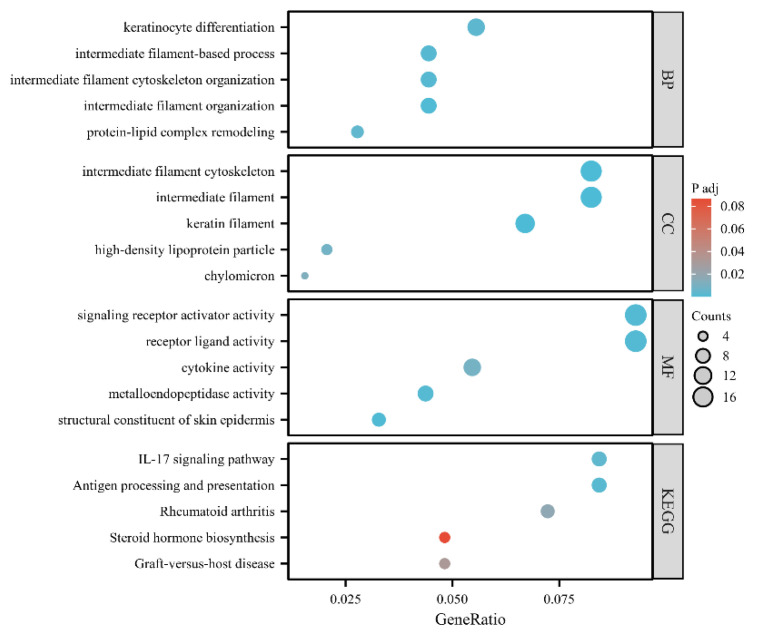
GO analysis and KEGG pathways for DEGs.

GO and KEGG enrichment analyses revealed the potential biological functions of DEGs. The top five GO items (biological processes (BP); cell component (CC); molecular function (MF)) and top five KEGG pathways are shown in Figure 4. The BP terms of the DEGs were primarily related to the extracelluar matrix. The top three BP terms included positive regulation of intermediate filament organization (8 genes, *P*_adj_ = 6.75 × 10^−4^), keratinocyte differentiation (10 genes, *P*_adj_ = 3.00 × 10^−3^), and protein–lipid complex remodelling (5 genes, *P*_adj_ = 3.67 × 10^−3^) (Figure 3e). The top five KEGG pathways were related to immune and inflammatory responses, including antigen processing and presentation (seven genes, *P*_adj_ = 0.0019), IL-17 signalling pathway (seven genes, *P*_adj_ = 0.0033), rheumatoid arthritis (six genes, *P*_adj_ = 0.018), graft-versus-host disease (four genes, *P*_adj_ = 0.032), and steroid hormone biosynthesis (four genes, *P*_adj_ = 0.086) (Figure 4). 

To choose the most correlated modules, the Gene Significance (GS) and Module Membership (MM) were calculated [20]. Among the modules, the brown module had the highest correlation coefficient (cor = 0.61, *p* < 0.001) between the gGS and MM (Figure S2). 

### 3.5. Hub Genes

**Figure 5:**
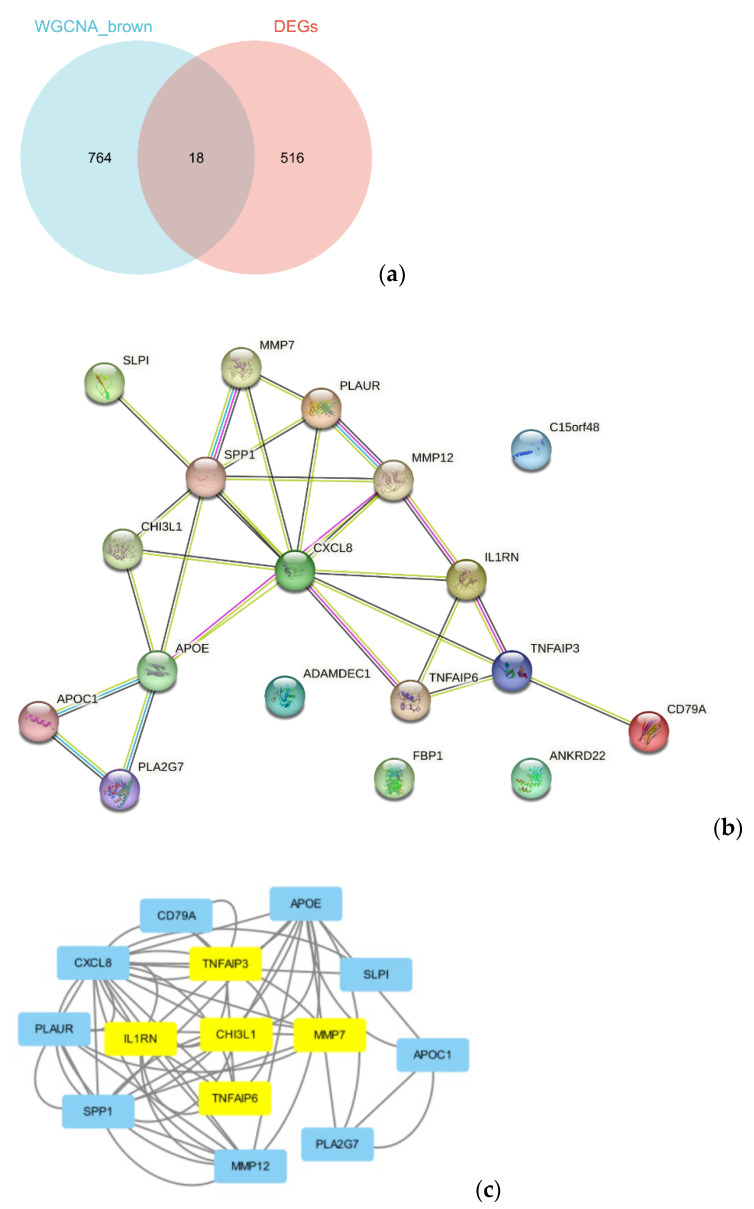
DEG co-expression network. (**a**) A Venn diagram displays the 18 common genes between the brown module and the total DEGs. (**b**) DEGs co-expression network of the brown module (**c**) Genes with top 5 MCODE score. The MCODE score reflects the density of the node and surrounding nodes. The yellow boxes represent the hub genes.

**Figure 6:**
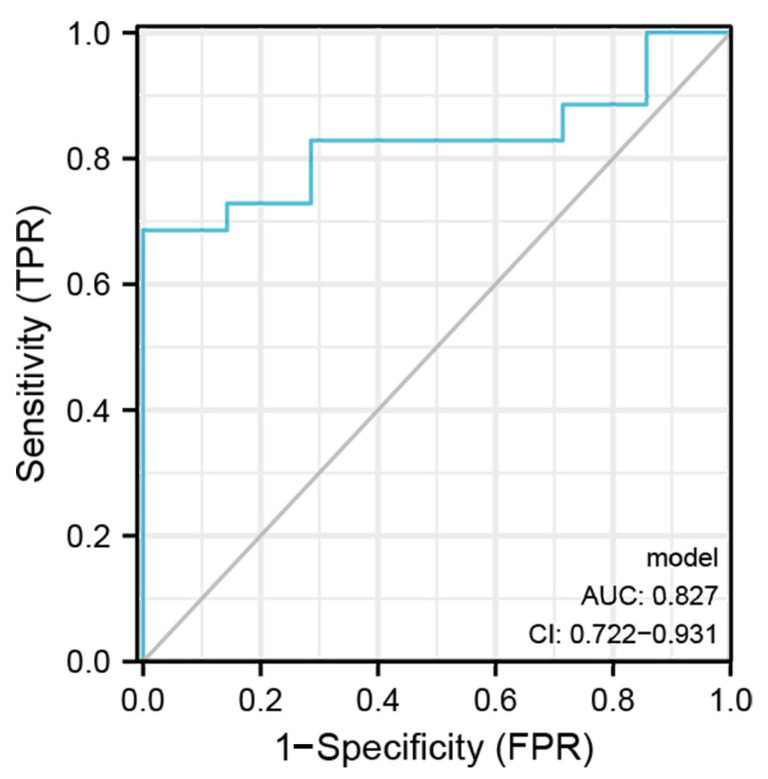
ROC curve of each of the 5 hub genes.

**Figure 7:**
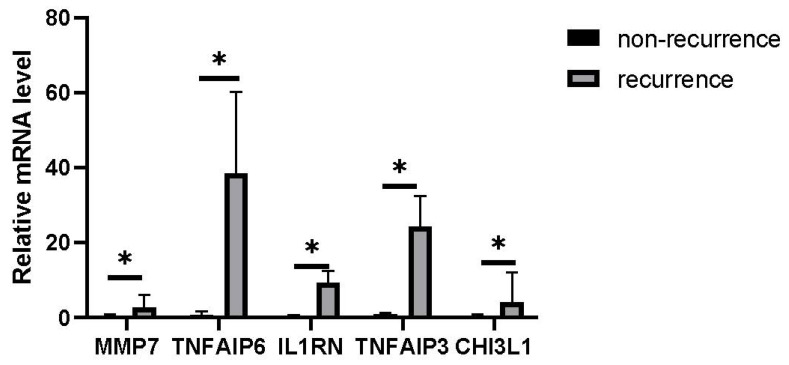
Differences in the expression of CHI3L1, IL1RN, MMP7, TNFAIP3, and TNFAIP6 between recurrent keloid and non-recurrent keloid tissues. (Note: * compares the recurrent keloid tissues with non-recurrent keloid tissues. *p* < 0.05).

The brown module contained 18 DEGs, all of which were upregulated (Figure 5a). Based on them, a DEG co-expression network was generated (Figure 5b). The network had 54 edges and 14 nodes. The top five hub genes are shown in Figure 5c. *CHI3L1* (Chitinase-3-Like Protein 1, log2FC = 2.48, *p* = 0.003), *IL1RN* (Interleukin 1 Receptor Antagonist, log2FC= 3.46, *p* = 4.71 × 10^−8^), *MMP7* (Matrix Metallopeptidase 7, log2FC = 2.62, *p* = 0.004), *TNFAIP3* (TNF Alpha Induced Protein 3, log2FC = 1.55, *p* = 8.34 × 10^−6^), and *TNFAIP6* (TNF Alpha Induced Protein 6, log2FC= 2.20, *p* = 1.31 × 10^−4^) were the top five hub genes with the highest MCODE score in the crucial gene cluster. ROC curve analysis (Figure 6) indicated the combination of hub genes (AUC = 0.776). The qRT-PCR experiment was used for detecting the differential expression of five hub genes (*CHI3L1*, *IL1RN*, *MMP7*, *TNFAIP3*, and *TNFAIP6*) in recurrent and non-recurrent keloid tissues. The result showed that compared to those in non-recurrent tissues, *CHI3L1*, *IL1RN*, *MMP7*, *TNFAIP3*, and *TNFAIP6* were found to be upregulated in the recurrent tissues, and the difference was statistically significant (*p* < 0.05) (Figure 7).

### 3.6. Results of Immune Infiltration

**Figure 8:**
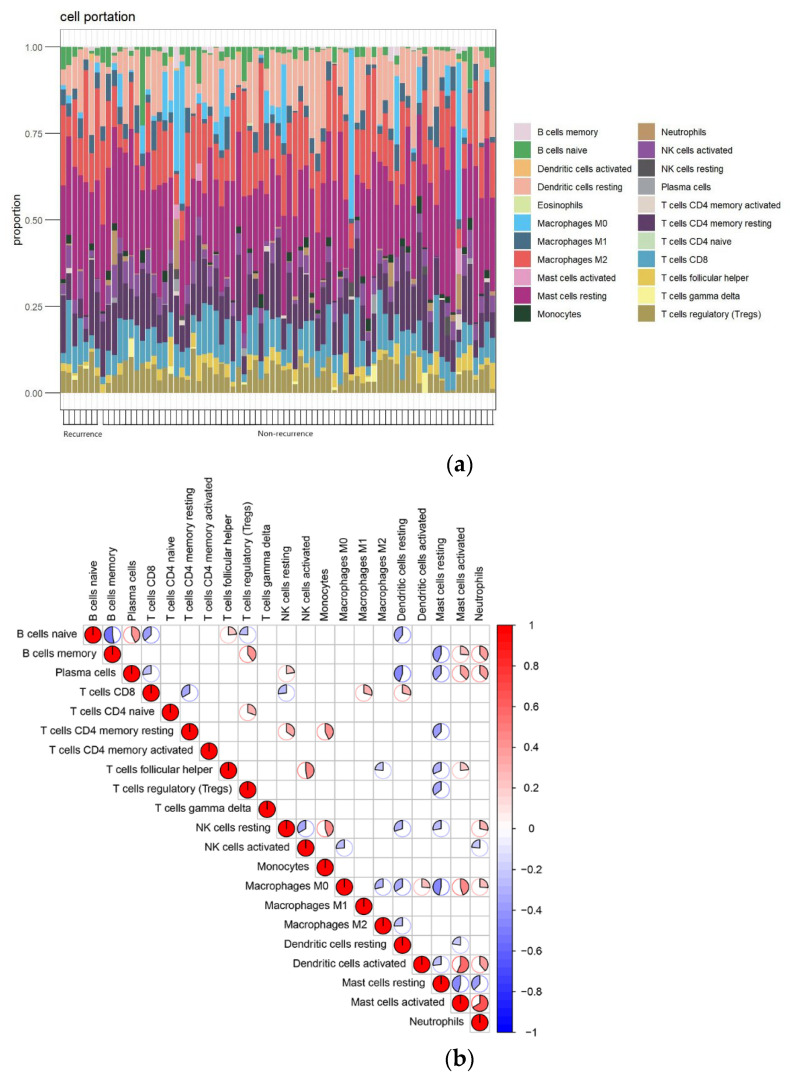

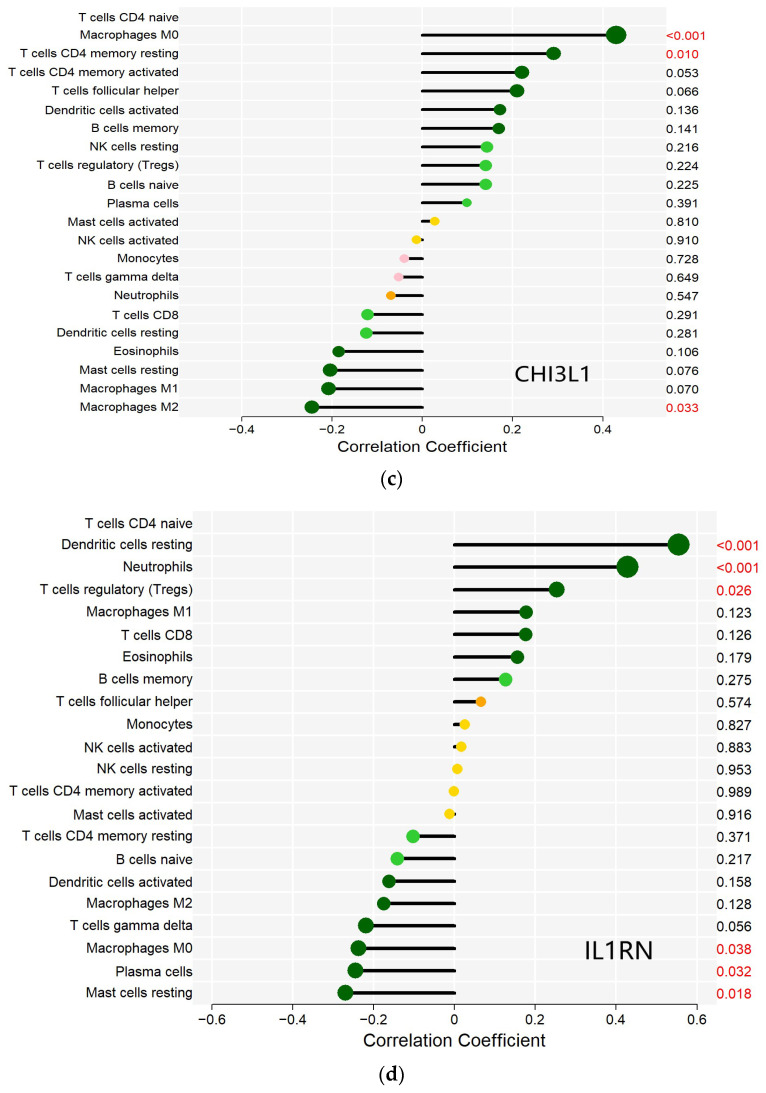

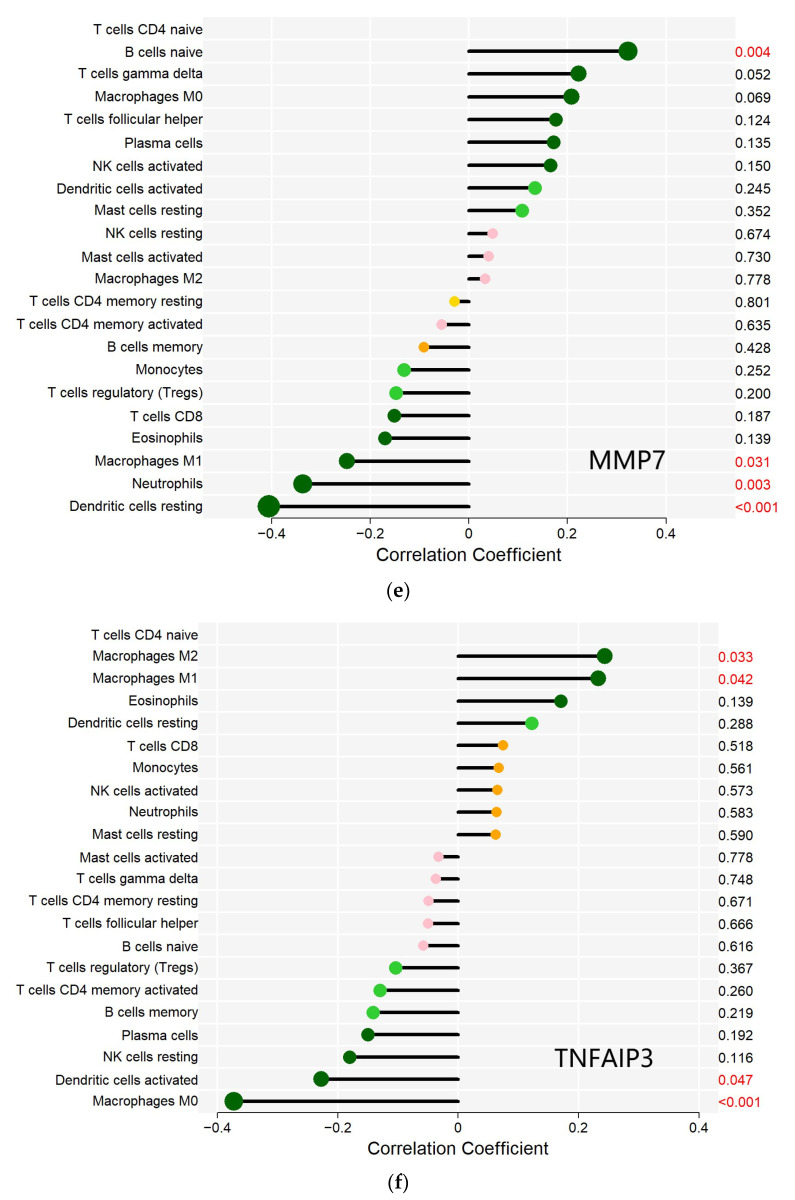

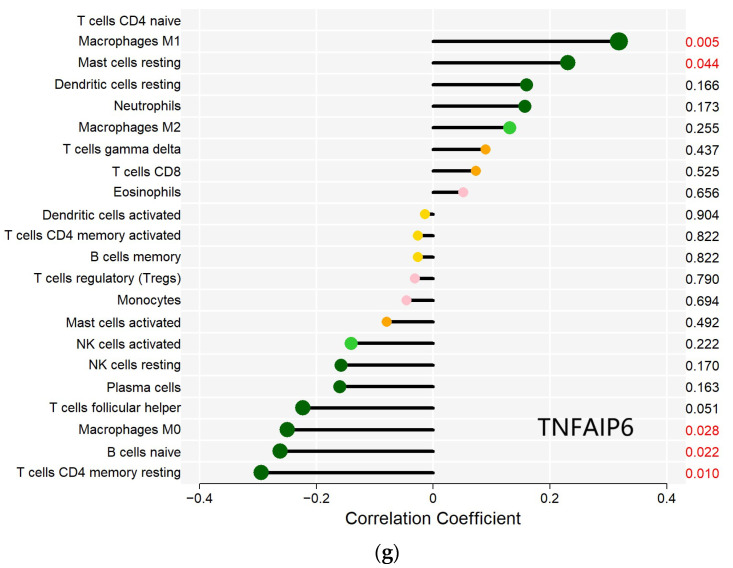
Immune infiltration analysis of 22 cells. (**a**) Correlation analysis of different immune cells. Red represents a positive correlation and blue represents a negative correlation. (**b**) Heatmap of the proportions of 22 immune cells in all samples. (**c**) Analysis of the correlation between CHI3L1 and immune cells. (**d**) Analysis of the correlation between IL1RN and immune cells. (**e**) Analysis of the correlation between MMP7 and immune cells. (**f**) Analysis of the correlation between TNFAIP3 and immune cells. (**g**) Analysis of the correlation between TNFAIP6 and immune cells. Red numbers indicate values less than 0.05.

We analyzed the immune infiltration in all samples. According to the spectrum of immune cells (Figure 8a), we found that mast cells and macrophages M2 and T cells were dominant in keloid tissues. The correlation analysis of the immune cells was also performed (Figure 8b), and the result showed that B cell memory was positively associated with regulatory T cells (Tregs) and negatively associated with naïve B cells and resting mast cells in keloid tissue. Tregs and mast cells resting had a negative correlation. Dendritic cells were activated and macrophages M0 were negatively correlated with activated mast cells. Neutrophils were positively associated with B cell memory, plasma cells, macrophages M0, and activated mast cells, and negatively associated with resting mast cells. As suggested by the correlation analysis between hub genes and immune cells, *CHI3L1* showed a positive correlation with macrophages M0 and T cells CD4 memory resting, and a negative correlation with macrophages M2 (Figure 8c). *IL1RN* showed a positive correlation with resting dendritic cells, neutrophils, and Tregs, but a negative correlation with macrophages M0, plasma cells, and resting mast cells (Figure 8d). *MMP7* was positively correlated with naïve B cells and negatively correlated with macrophages M1, neutrophils, and resting dendritic cells (Figure 8e). *TNFAIP3* was positively correlated with macrophages M1 and M2, and negatively correlated with activated dendritic cells and macrophages M0 (Figure 8f). *TNFAIP6* was positively correlated with macrophages M1 and resting mast cells, and negatively correlated with macrophages M0, naïve B cells, and T cells CD4 memory resting (Figure 8g). 

### 3.7. Discussion

In this study, we analyzed 534 DEGs between 70 non-recurrent and 7 recurrent keloid tissue samples and 18 gene co-expression modules associated with recurrent keloids. The DEGs were significantly enriched in keratinocyte differentiation, intermediate filament, receptor ligand activity, metalloendopeptidase activity, cytokine activity, IL-17 signalling pathway, etc. Previous studies have demonstrated that age, sex, etiology, and other common clinical features are not reliable predictors of keloid recurrence [21,22]. Thus, these genes may facilitate the design of new and efficient predictive tools for recurrent keloids. 

Few studies have been conducted to identify biomarkers of keloid recurrence. In a histopathological study, it is found that abnormal proliferation of vessels, activation of fibroblasts, and infiltration of inflammatory cells are hallmarks of keloid growth and recurrence [23]. Mast cells, macrophages, T cells, and other immune cells are associated with keloid formation [2,3]. These results suggest that abnormal immune responses are intimately implicated in keloid recurrence after surgery. In the present study, the hub genes (*CHI3L1*, *IL1RN*, *MMP7*, *TNFAIP3*, and *TNFAIP6*) were mainly involved in the regulation of inflammatory response, IL-17 signalling pathway, and TNF signalling pathway, which is consistent with previous findings in keloids. Previous studies suggested that IL-17 plays a significant role in keloid fibrosis [24,25]. Also, Messadi et al. found that the upregulation of TNF-alpha is related to the activation of NKĸB in keloids [26]. Therefore, we hypothesize that immune-related genes might play a major role in keloid recurrence. 

We constructed a DEG co-expression network based on the 769 DEGs in the brown module and identified five hub genes (*CHI3L1*, *IL1RN*, *MMP7*, *TNFAIP3*, and *TNFAIP6*). The combination of these five hub genes was further proven to have a strong ability to predict keloid recurrence, with an AUC of 0.776. Finally, we validated the expression of hub genes in 10 keloid samples. Compared to those in the non-recurrent tissues, five hubs were upregulated in the recurrent tissues, and the difference was statistically significant (*p* < 0.05).

*CHI3L1* (Chitinase-3-Like Protein 1), a secreted glycoprotein, acts in diseases associated with extracellular matrix (ECM) remodelling and inflammation, Th2-related inflammation, and interleukin-13-induced inflammatory response [27]. In keloid diseases, *CHI3L1* can activate fibroblasts and promote collagen deposition via TGF-β signalling pathways [28]. In chronic wounds, such as healing wounds with diabetic foot ulcers, the fibroblasts overexpress *CHI3L1*, *MMPs*, and *TNFAIP6* [29], similar to the hub genes in this study. A study has shown that when fibroblast *CHI3L1* expression is knocked down in an orthotopic mouse model of breast cancer, the level of activated (α-SMA–expressing) fibroblasts drops [30]. 

*IL1RN* is a member of the interleukin 1 cytokine family, also named interleukin 1 receptor antagonist, which modulates a variety of interleukin 1 related inflammatory responses. Tilg, H. et al. have treated several plasma samples with IL-6, finding that *IL1RN* expression is rapidly increased [31]. As the upregulation of IL-6 plays a role in keloid scars, we infer that *IL1RN* participates in the deposition of collagen via IL-6 signalling pathways [32]. However, in hypertrophic scars, the expression of *IL1RN* gradually decreases during wound healing compared to that in normal scars [33]. The mechanism of *IL1RN* in the recurrence of keloid needs more explorative research. 

*MMP7* (Matrix Metallopeptidase 7), a member of the matrix metalloproteinases (MMPs), is involved in normal physiological processes, such as the breakdown of the extracellular matrix, tissue rebuilding, and embryonic development. M. Fujiwara et al. investigated the MMPs in keloids and found that the levels of MMP-1 and MMP-2 are 6-fold and 2.4-fold greater than those fibroblasts in normal skin tissue, respectively, and the upregulation of MMP2 is associated with the signalling of TGF-β [34], a classical pathway in pathogenesis in keloids. It is reasonable to hypothesize that MMP7 may interfere with the construction of extracellular matrix components, resulting in a higher risk of keloid recurrence.

*TNFAIP3* and *TNFAIP6* are important factors in the TNF-alpha signalling pathway that can be rapidly induced by the tumor necrosis factor (TNF) [35,36]. As downstream genes of TNF, *TNFAIP3* and *TNFAIP6* may be overexpressed to activate the TNF-α signalling pathway. TNF-α promotes cell proliferation by activating the nuclear factor (NF)-κB [37], and several studies have shown that NF-κB participates in keloid formation [26,38]. TNF is also linked to many other immune-related skin diseases [39,40]. The TNF blockade has been validated as a therapeutic strategy for psoriasis [41]. In this study, the TNF signalling pathway was significantly activated in keloid recurrence tissues, suggesting that the TNF-alpha may be a target to prevent the recurrence of keloids. 

In this study, we assessed the infiltration of immune cells into keloid tissues and found that mast cells, T cells, and macrophages are important components of the keloid immune microenvironment. Mast cells promote fibroblast proliferation via VEGF, IL-4, and basic fibroblast growth factor (bFGF), thereby stimulating type I collagen synthesis [42]. By releasing TGF-β and platelet-derived growth factor-CC (PDGF-CC), macrophages play a pivotal role in stimulating the conversion of fibroblasts into myofibroblasts. This process, in turn, facilitates collagen deposition and contributes to the development of keloid formation. [43]. In a recent study, the levels of macrophage M0 infiltration vary among keloid tissues [15]. These findings indicate that the functions of these genes are closely related to the immune and inflammatory mechanisms. 

There are several limitations to this study. First, the keloid tissue samples were predominantly obtained from the most visibly inflamed regions, and the heterogeneity in keloid tissue may interfere with the prediction effect of biomarkers on postoperative recurrence. Future research should encompass a more comprehensive assessment of genes and proteins across multiple areas within the keloid. Second, the postoperative recurrence definition in this study relied on one year follow-up period, but if we can follow up for two years, the judgment of recurrence will be more accurate. In the future, we will expand the sample size and extend the follow-up time to verify the results of this study. Third, we did not perform gain-of-function and loss-of-function analyses to explore the role of the hub genes in keloid recurrence. The underlying molecular mechanism of these hub genes needs substantiation in future experimental research. Fourth, we did not perform single-cell RNA-seq or fluorescence in situ hybridization to explore the location of hub genes.

## 4. Conclusions

This study is the first to employ WGCNA to identify hub genes associated with keloid recurrence. These hub genes were enriched in immune and inflammatory responses. Five hub genes were linked to keloid recurrence, including *CHI3L1*, *IL1RN*, *MMP7*, *TNFAIP3*, and *TNFAIP6*. 

This study elucidates the underlying mechanisms of keloid recurrence from a molecular biology perspective, providing a foundation for predicting and preventing keloid recurrence in future clinical applications.

## Figures and Tables

**Table 1:** Basic clinical information comparison between non-recurrent and recurrent samples.

Characteristics	Nonrecurrence	Recurrence	*p*	Univariable		Multivariable	
				OR (95%CI)	*p*	OR (95% CI)	*p*
N	70	7					
Age, years	28.0 (23.2–42.0)	25.0 (21.5–28.0)	0.121	0.9 (0.8, 1.0)	0.149	1.0 (0.9, 1.1)	0.625
Duration, months	36.0 (24.0–60.0)	36.0 (30.0–48.0)	0.612				
VSS	11.0 (10.0–11.0)	11.0 (10.0–11.0)	0.8				
DLQI	13.0 (11.0–15.0)	14.0 (12.5–17.0)	0.222				
Sex			0.011				
Male	35 (50.0%)	7 (100.0%)		1		1	
Female	35 (50.0%)	0 (0.0%)		0.0 (0.0, Inf)	0.995	0.0 (0.0, Inf)	0.997
Site			0.506				
Earlobe	10 (14.3%)	0 (0.0%)					
Face	16 (22.9%)	3 (42.9%)					
Trunk	41 (58.6%)	4 (57.1%)					
Vulva	3 (4.3%)	0 (0.0%)					
Cause			0.007				
Surgery	12 (17.1%)	1 (14.3%)		1		1	
Trauma	7 (10.0%)	0 (0.0%)		0.0 (0.0, Inf)	0.998	0.0 (0.0, Inf)	0.998
Inflammation (acne)	17 (24.3%)	6 (85.7%)		4.2 (0.4, 39.9)	0.207	1.5 (0.1, 16.8)	0.741
Unknown	34 (48.6%)	0 (0.0%)		0.0 (0.0, Inf)	0.995	0.0 (0.0, Inf)	0.997
Postoperative radiotherapy			0.516				
No	4 (5.7%)	0 (0.0%)					
Yes	66 (94.3%)	7 (100.0%)					

Continuous variables are expressed as median (inter-quartile range), and categorical variables are expressed as numbers and percentages. For continuous variables, the Kruskal–Wallis rank sum test was used. If the count variable had a theoretical number < 10, Fisher’s exact probability test was used. CI: confidence interval, OR: odds ratio.

**Table 2:** Top 10 DEGs between recurrent and non-recurrent samples.

Gene Symbol	Official Full Name	log_2_FoldChange	*p*-Value
**Upregulated**			
IGLV5-37	Immunoglobulin Lambda Variable 5-37	4.620669009	0.047323941
LINC01093	Long Intergenic Non-Protein Coding RNA 1093	4.305514686	0.015120619
AL591468.1	Long Non-Coding RNA	4.019911778	0.011245769
AC007991.2	Long Non-Coding RNA	3.926654734	0.000453876
MT1H	Metallothionein 1H	3.773067315	2.86 × 10^−5^
AC079753.1	Long Non-Coding RNA	3.762199714	0.00352934
AC106865.1	Long Non-Coding RNA	3.725765246	6.06 × 10^−6^
FFAR3	Free Fatty Acid Receptor 3	3.717308852	0.000687165
C15orf48	Chromosome 15 Open Reading Frame 48	3.632543784	7.66 × 10^−7^
FBP1	Fructose-Bisphosphatase 1	3.597862858	1.01 × 10^−8^
**Downregulated**			
LHX9	LIM Homeobox 9	−5.49854215	2.10 × 10^−5^
KRTAP16-1	Keratin-Associated Protein 16-1	−5.21285097	0.020551069
KRTAP3-3	Keratin Associated Protein 3-3	−4.97829611	0.04444846
KRTAP3-2	Keratin Associated Protein 3-2	−4.80908224	0.0252855
SLC18A3	Solute Carrier Family 18 Member A3	−4.68579157	0.023883896
PSG5	Pregnancy-Specific Beta-1-Glycoprotein 5	−4.41453451	7.97 × 10^−5^
AC011591.2	Long Non-Coding RNA	−4.35433212	0.000589416
RETN	Resistin	−4.16858124	0.000426338
KRT83	Keratin 83	−4.16466678	0.019938752
KRTAP1-5	Keratin Associated Protein 1–5	−4.16368439	0.021765483

## Data Availability

All sequence data used in the analyses were deposited in Sequence Read Archive (SRA) (http://www.ncbi.nlm.nih.gov/sra, accessed on 22 August 2023) under BioProject PRJNA813172.

## Supplementary Materials

The following supporting information can be downloaded at: https://www.mdpi.com/article/10.3390/jcm12175511/s1, Figure S1: Principal component analysis generated by all differential expression genes for all samples; Figure S2: The correlation of the gene significance for recurrence and module membership in brown module; Table S1: Primer sequence of qRT-PCR; Table S2: All DEGs between recurrence samples and non-recurrence samples under the threshold of *p* < 0.05 and the |log2FC| > 1.5; Table S3: The RIN of all tissue samples; Supplementary Explanation: The detailed protocol of RNA extraction and library construction.
