# Supplementary material for: Tissue RNA Sequencing Reveals Novel Biomarkers Associated with Postoperative Keloid Recurrence
Source: J Clin Med. 2023 Aug 25;12(17):5511. doi: 10.3390/jcm12175511 (PMC10488753; doi:10.3390/jcm12175511)
Supplement: Supplementary file 1 [file jcm-12-05511-s001.zip › Supplementary Explanation.pdf]

RNA extraction and library construction

The RNA-Seq library was prepared by the NEBNext® Ultra™ RNA Library Prep Kit for Illumina (E7530S, NEB, USA). RNA integrity was evaluated by the RNA Nano 6000 Assay Kit of the Bioanalyzer 2100 system (Agilent Technologies, CA, USA). 1 μg RNA of each sample was input for preparation. Initially, poly-T oligo-attached magnetic beads was utilized to extract mRNA from total RNA, followed by fragmentation employing divalent cations in First Strand Synthesis Reaction Buffer (5X) under elevated temperature. Random hexamer primers and M-MuLV Reverse Transcriptase were employed to initiate the synthesis of first strand cDNA. Following this, RNaseH was utilized to degrade the RNA template. After the first strand cDNA synthesis, the second strand cDNA was synthesized using DNA Polymerase I and dNTP. To achieve this, any remaining overhangs were modified into blunt ends through the activities of exonucleases and polymerases. Following the adenylation of the 3' ends of DNA fragments, adaptors featuring a hairpin loop structure were ligated to facilitate hybridization. To isolate cDNA fragments of specific length, ranging from 250 to 300 base pairs, the library fragments were purified using the AMPure XP system from Beckman Coulter, located in Beverly, USA. Afterwards, a 15-minute incubation at 37°C was performed, with 3 μl of USER enzyme (obtained from NEB, USA) added to the size-selected, adaptor-ligated cDNA. This was followed by a 5-minute incubation at 95°C before proceeding with PCR. Next, PCR was conducted using Phusion High-Fidelity DNA polymerase, along with Universal PCR primers and Index (X) Primer. Finally, the PCR products were purified using the AMPure XP system, and the quality of the library was evaluated on the Agilent Bioanalyzer 2100 system. The RNA integrity number of all tissue samples were shown in the Table S3.
