# Supplementary material for: Tissue RNA Sequencing Reveals Novel Biomarkers Associated with Postoperative Keloid Recurrence
Source: J Clin Med. 2023 Aug 25;12(17):5511. doi: 10.3390/jcm12175511 (PMC10488753; doi:10.3390/jcm12175511)
Supplement: Supplementary file 1 [file jcm-12-05511-s001.zip › Supplementary Figure S1.pdf]

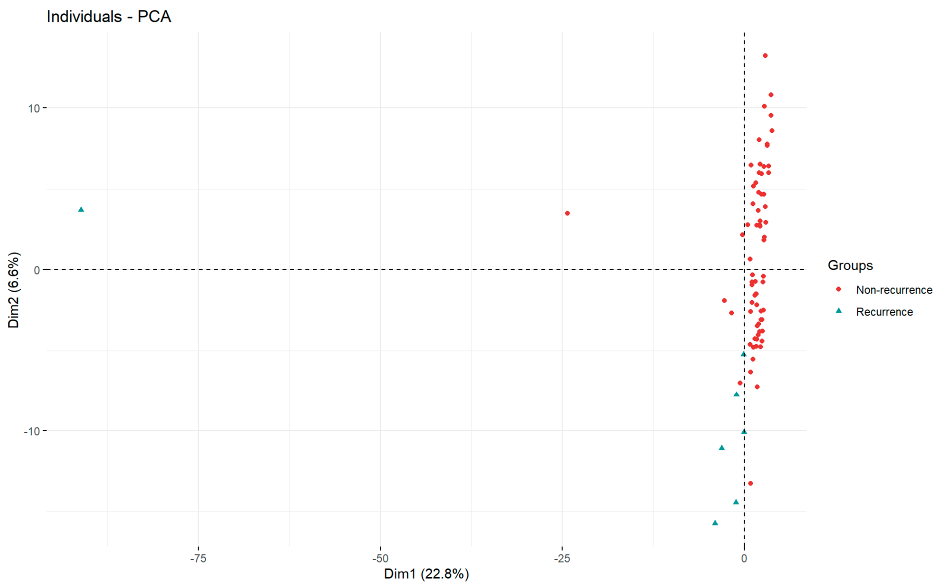

Figure S1. Principal component analysis generated by all differential expression genes for all samples.
