# Supplementary material for: Tissue RNA Sequencing Reveals Novel Biomarkers Associated with Postoperative Keloid Recurrence
Source: J Clin Med. 2023 Aug 25;12(17):5511. doi: 10.3390/jcm12175511 (PMC10488753; doi:10.3390/jcm12175511)
Supplement: Supplementary file 1 [file jcm-12-05511-s001.zip › Supplementary Figure S2.pdf]

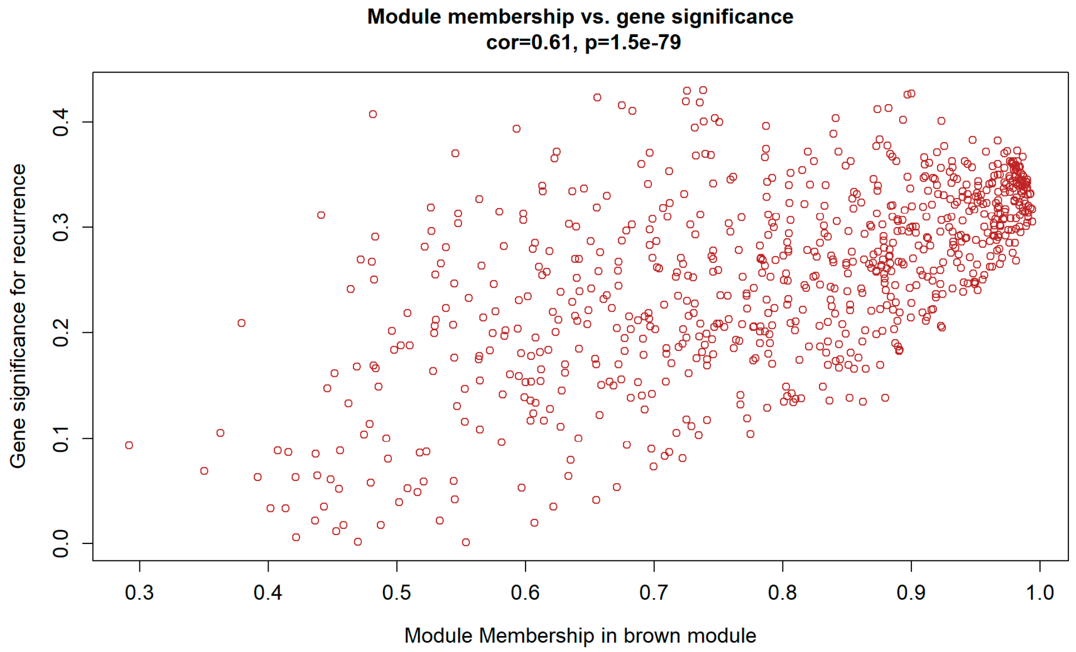

Figure S2. The correlation of the gene significance for recurrence and module membership in brown module.
