# Supplementary material for: Tissue RNA Sequencing Reveals Novel Biomarkers Associated with Postoperative Keloid Recurrence
Source: J Clin Med. 2023 Aug 25;12(17):5511. doi: 10.3390/jcm12175511 (PMC10488753; doi:10.3390/jcm12175511)
Supplement: Supplementary file 1 [file jcm-12-05511-s001.zip › Supplementary Information.pdf]

Supplementary Information

Tissue RNA Sequencing reveals novel biomarkers associated with postoperative keloid recurrence

Kehui Ren[1#], Yanqiu Tang[1#], Xufeng Yin[1], Yunning Yang[1], Fang Fang[2], Bingrong Zhou[1]*, Wenbo Bu[2]*

[1] Department of Dermatology, The First Affiliated Hospital of Nanjing Medical University, Nanjing, China

[2] Department of Dermatologic surgery, Dermatology Hospital of Chinese Academy of Medical Sciences, Nanjing, China

Supplementary Figure S1. Principal component analysis generated by all differential expression genes for all samples.

Supplementary Figure S2. The correlation of the gene significance for recurrence and module membership in brown module.

Supplementary Table S1. Primer sequence of qRT-PCR.

Supplementary Table S2. All DEGs between recurrence samples and non-recurrence samples under the threshold of $p<0.05$ and the $|\log2FC| > 1.5$.

Supplementary Table S3. The RIN of all tissue samples.

Supplementary Explanation. The detailed protocol of RNA extraction and library construction.

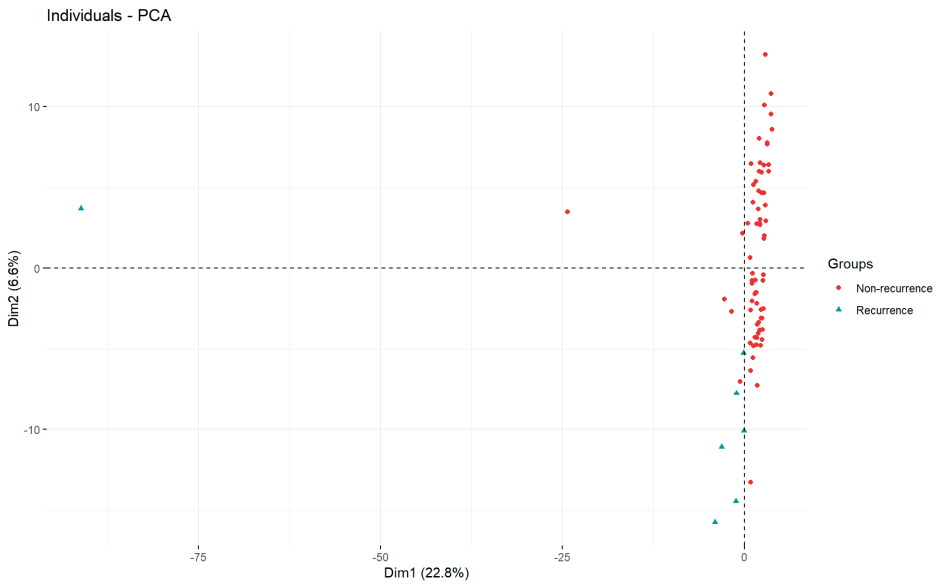

Figure S1. Principal component analysis generated by all differential expression genes for all samples.

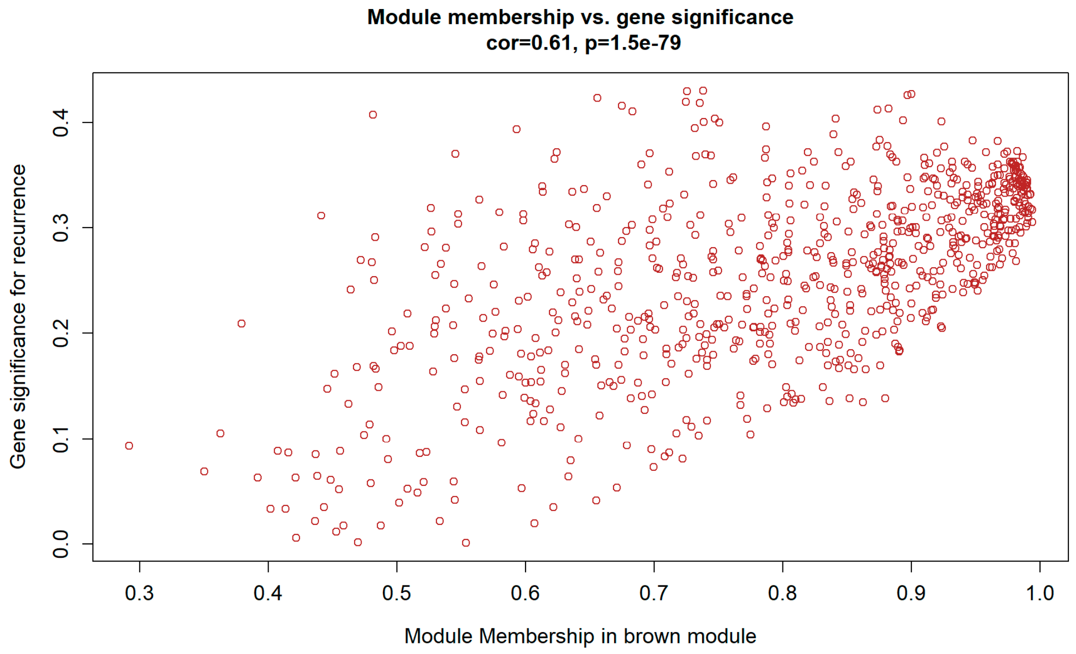

Figure S2. The correlation of the gene significance for recurrence and module membership in brown module.

Table S1. Primer sequence of qRT-PCR.

	Primer	Sequence (5'- 3')
FERMT3	The sense primer	CATCGACTCGTCATGGGAGC
	The antisense primer	GGTCTGACCAGTCCTGCTTG
VEGFA	The sense primer	GGAGGGCAGAATCATCACGA
	The antisense primer	GCTCATCTCTCCTATGTGCTGG
LCP1	The sense primer	GTCTGCCTGTTCTGGACCTC
	The antisense primer	TGGCAGGGCATACACTCTTG
CD86	The sense primer	CCAGACCACATTCCTTGGATTAC
	The antisense primer	CTCTTTTCTTGGTCTGTTCACTCTC
APBB1IP	The sense primer	AGAAATCCTGGAAAAGGCGCT
	The antisense primer	CAAAGCAATAGTCAGTGGGCG
